# The Year's Work of the Medical Charities of London

**Published:** 1890-05-31

**Authors:** 


### The Year's Work of the Medical Charities of London.

The voluntary hospitals and medical charities of London during the year ended the 31st of December, 1889, relieved one million one hundred thousand patients, at a cost of £571,483, against an ordinary income of only £496,759, being a difference of nearly seventy-five thousand pounds on the year's work. Of eight thousand and sixty-three available beds only six thousand and thirty were constantly occupied throughout the year, leaving one-fourth of the whole number empty, many of which might have been filled had funds been forthcoming to defray the cost. There is evidence that the same system of starvation is applied to the voluntary hospitals all over London, though it is practised with more definiteness in some metropolitan districts than in others.

In the district of Newington and South London there are ten hospitals and one convalescent home (containing fifteen hundred and sixty-three beds, of which one thousand and twenty-five were constantly occupied), and eleven dispensaries. These institutions relieved during the year thirteen thousand four hundred in-patients and one hundred and twenty-eight thousand out-patients, at a total cost of eighty-one thousand pounds. The total income was less than eighty thousand pounds in round numbers, of which only thirty-three thousand was supplied by voluntary contributions, so that the inhabitants of South London give at the present time very much less than half the sum required to maintain the existing medical charities in efficiency. Yet South Londoners have frequently complained of late that there is not sufficient hospital accommodation in their midst, and that they ought to have very many more beds than at present. It is our duty to point out, however, that the inhabitants of Tooting, Balham, Streatham, Brixton, Southwark, Camberwell, Lewisham, Blackheath, Woolwich, and other places, before they are justified in demanding new institutions, must show a more liberal spirit towards those they already possess.

In the City and East Central District, which comprises the City, St. Luke's, Shoreditch, Finsbury, and Clerkenwell, the medical charities contain five hundred and eighty-three beds, of which four hundred and twenty-seven were constantly occupied in 1889, at a cost of upwards of forty-nine thousand pounds. The total ordinary income amounted to less than thirty-nine thousand pounds, leaving a deficiency of ten thousand pounds. There are nine hospitals and seven dispensaries in the district, and this ten thousand pounds ought to be promptly forthcoming.

In the district of Westminster, which includes Westminster city and its liberties, there are twelve hospitals and six dispensaries, which collectively relieved one hundred and sixty thousand persons, of whom nine thousand two hundred and ninety were in, and one hundred and fifty thousand were out-patients. The hospital beds available amounted to nine hundred and sixty-four, of which seven hundred and sixty-seven were constantly occupied. The total expenditure on behalf of the medical charities exceeded eighty thousand pounds, whilst less than sixty-four thousand pounds of income was available, of which, however, more than two-thirds, or £43,000 was supplied by the present inhabitants of Westminster.

In St. Marylebone and West Central District, which includes St. John's Wood, Bloomsbury, and Holborn, there are twenty hospitals and institutions admitting in-patients, which contained twelve hundred and fifty-nine beds, whilst only nine hundred and forty-nine were constantly occupied throughout 1889. In addition to the hospitals there are six dispensaries, and altogether upwards of one hundred and forty-three thousand persons were relieved, of which eight thousand eight hundred were in, and one hundred and thirty-four thousand were out-patients. The total expenditure of the medical charities in 1889 approached ninety-two thousand pounds; whilst the total ordinary income amounted to less than eighty thousand pounds, of which rather less than two-thirds was subscribed by the inhabitants.

In Kensington and West District, which includes Paddington, Bayswater, Chelsea, Brompton, and Hammersmith, and which extends to Brentford and Ealing, there are thirteen hospitals (containing 1,570 beds, of which upwards of thirteen hundred were constantly occupied), and twelve dispensaries. One hundred and sixty-three out-patients, and nearly fourteen thousand five hundred in-patients, were relieved at a total cost of nearly one hundred and nineteen thousand pounds. The total ordinary income amounted to less than one hundred and one thousand pounds, leaving a deficiency of seventeen thousand pounds.

Islington and the North-west District contains nine hospitals and seven dispensaries, which collectively relieved nearly one hundred and twenty-nine thousand persons, of whom upwards of seven thousand were in-patients, and one hundred and twenty-one thousand out-patients. The expenditure on medical relief exceeded fifty-seven thousand pounds, and the ordinary income almost reached fifty-one thousand pounds. A very creditable fact, and one which we imagine may be due in a measure to the circumstance that North London has set an example to other districts, by placing a great general hospital in its immediate centre, and has so become self-contained and self-supporting.

In the districts of Stratford and the East-end, which includes the Tower Hamlets and Hackney, there are five hospitals and one convalescent institution (containing upwards of thirteen hundred beds, of which upwards of one thousand were occupied), and six dispensaries. Nearly a quarter of a million of persons were relieved by these charities, at a cost of about ninety-three thousand pounds, of which eighty-three thousand pounds was subscribed, leaving a deficiency of ten thousand pounds. Having regard to the poverty of the district, this result is remarkable, and may be due, as in the case of Islington, to the fact that the people of West Ham have resolved to establish a general hospital in their midst, and to support it themselves.

We have very briefly sketched the work of the hospital and medical charities of London during 1889, and have shown to what extent that work has been supported with funds. It will be seen that in every district there is need for a more active interest in the work of our hospitals. We are convinced, however, that if the ministers of all denominations really exert themselves, at least £75,000, *i.e.*, the whole deficiency, will this year be forthcoming as the result of the Hospital Sunday collections. But if the preacher does not feel called upon to preach a special sermon on Hospital Sunday in the cause of suffering humanity how can he expect his people to realise or to believe that it is necessary for them to give a special subscription to the hospitals on that day?

